# Association between oxidative balance score and kidney stone in United States adults: analysis from NHANES 2007-2018

**DOI:** 10.3389/fphys.2023.1275750

**Published:** 2023-11-03

**Authors:** Runjiang Ke, Youhua He, Chaohao Chen

**Affiliations:** Department of Urology, The Second Affiliated Hospital and Yuying Children’s Hospital of Wenzhou Medical University, Wenzhou, Zhejiang, China

**Keywords:** oxidative balance score, kidney stones, NHANES, antioxidants, pro-oxidants

## Abstract

**Purpose:** To investigate the relationship between the Oxidative Balance Score (OBS) and kidney stone risk using NHANES 2007-2018 data, and to explore potential mechanisms and population-specific effects.

**Materials and methods:** Data from the NHANES 2007-2018 were analyzed. OBS was calculated based on 16 dietary components and 4 lifestyle components. Multivariate logistic regression was employed to investigate the relationship between OBS and kidney stone. Further stratified analyses were conducted to examine the associations across different subgroups.

**Results:** A total of 19,799 participants were included in the study. There was a consistent inverse association between OBS and the risk of kidney stones (OR = 0.97; 95% CI: 0.96–0.99). After dividing the participants into quartiles based on OBS, compared to the lowest quartile of OBS, the risk of kidney stones in the highest quartile of OBS was reduced by 33% (95% CI 0.50–0.89; *p* = 0.002). This association was consistent across both dietary and lifestyle OBS scores. The protective effect of OBS was notably pronounced among Non-Hispanic white and Other race groups, and among individuals with a higher level of education. However, the association was not significant among individuals with diabetes.

**Conclusion:** A higher OBS, indicating a balance skewed towards antioxidants, is associated with a reduced risk of kidney stones, especially among specific population subgroups. These findings underscore the potential role of oxidative balance in kidney stone pathogenesis and highlight the importance of considering individual and population-specific factors in future research and preventive strategies.

## 1 Introduction

The prevalence and incidence of kidney stones worldwide have been increasing over the past few decades. The burden of kidney stones on healthcare systems is substantial, with annual healthcare expenditures exceeding $2 billion in the United States alone (Thongprayoon et al., 2020). Kidney stone disease is a systemic disorder associated with chronic kidney disease, bone loss and fractures, increased risk of coronary artery disease, hypertension, type 2 diabetes, and metabolic syndrome (Sakhaee et al., 2012). The formation of kidney stones is a complex process regulated by metabolic changes in various substances, including oxalate, reactive oxygen species (ROS), hormones, and others. Among these, oxidative stress (OS) induced by ROS plays a key regulatory role in the formation of kidney stones (Xu et al., 2023). Animal and *in vitro* studies have confirmed that oxalate and calcium oxalate crystals can induce OS in renal epithelial cells, which is a critical factor leading to the formation of kidney stones (Umekawa et al., 2004; Zhu et al., 2019; Khan et al., 2021; Khan, 2014; W et al., 2021).

OS refers to a condition where the production of oxidants exceeds the body’s antioxidant defenses. Dietary intake is an important source of antioxidants and oxidants. However, the relationship between individual nutrient components and kidney stones remains contradictory due to conflicting study results and the complexity of the human diet. *In vitro* studies have confirmed that antioxidants such as vitamins C and E can protect against oxalate-induced OS and kidney damage (Thamilselvan et al., 2014; Jaturakan et al., 2017). However, excessive intake of vitamin C may increase urinary oxalate excretion and increase the risk of kidney stones (Ferraro et al., 2016; Jaturakan et al., 2017). Increasing fiber intake and maintaining balanced calcium intake can reduce the risk of kidney stone formation (Nirumand et al., 2018; Ferraro et al., 2020). Studies on the relationship between vitamin D and kidney stones are also inconsistent (Letavernier et al., 2016; Malihi et al., 2016; Ferraro et al., 2017; Bouderlique et al., 2019; Malihi et al., 2019; Taheri et al., 2019). Another study found that increasing the six major dietary sources of antioxidants (including vitamins A, beta-carotene, B6, C, E, and lycopene) did not significantly reduce the risk of kidney stone disease (Jian et al., 2021). In addition, lifestyle factors are also a major source of OS. It has been established that obesity is a risk factor for kidney stones (Milicevic et al., 2013; Sasaki et al., 2015; Lee et al., 2022a). Alcohol can cause OS damage to kidney tissues, leading to the formation of kidney stones (Jones et al., 2021). Whether smoking (including active and passive smoking) (Tamadon et al., 2013; Marić et al., 2019; Chen et al., 2021) and physical activity (Sorensen et al., 2014; Ferraro et al., 2015; Aune et al., 2018; Zhuo et al., 2019) significantly increase the risk of kidney stones is also inconclusive.

The Oxidative Balance Score (OBS) is a comprehensive measure that reflects both pro-oxidant and antioxidant exposures, mainly composed of dietary and lifestyle components (Hernández-Ruiz Á et al., 2022). A higher OBS indicates a higher antioxidant and lower pro-oxidant exposure, suggesting a lower level of OS. Previous studies have confirmed that a higher OBS can reduce the risk of some diseases, such as better blood sugar control (Golmohammadi et al., 2021), lower incidence of hypertension (Lee et al., 202b), as well as reducing depression (Liu et al., 2023), improving sleep quality (Lei et al., 2023), etc. However, the relationship between OBS and the risk of kidney stones has not been studied. Therefore, we used the NHANES 2007-2018 data to conduct a cross-sectional study on the relationship between OBS and kidney stones. This study could lead to a better understanding of the role of OS in kidney stone formation and potentially lead to new prevention strategies.

## 2 Materials and methods

### 2.1 Study population

**FIGURE 1:**
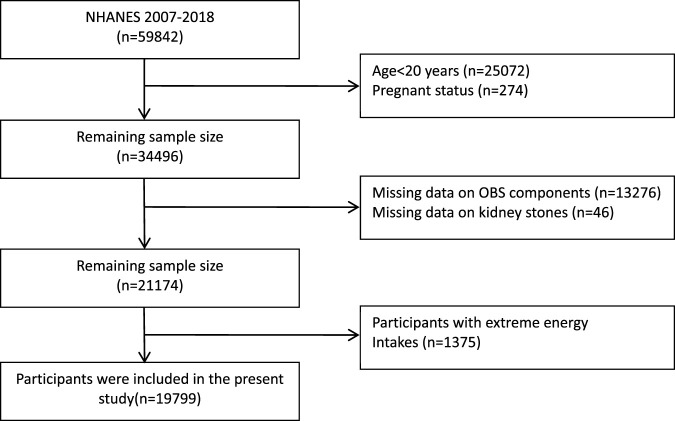
Flowchart of the study population. NHANES, National Health and Nutrition Examination Survey; OBS, oxidative balance score.

The National Health and Nutrition Examination Survey (NHANES), conducted every 2 years, is a program designed to assess the health and nutritional status of adults and children in the United States. All participants have provided informed consent. For our study, we incorporated data from five NHANES cycles spanning 2007–2018, initially involving 59,842 participants. We applied several exclusion criteria: 1) Individuals under 20 years of age (n = 25072); 2) pregnant individuals (*n* = 274); 3) individuals with missing OBS component data (*n* = 13276); 4) individuals with missing kidney stone questionnaire data (*n* = 46); 5) individuals with unreliable energy intake (for males: <800 kcal/d or >4,200 kcal/d, for females: <500 kcal/d or >3,500 kcal/d) (*n* = 1375) (Zhang et al., 2022). After applying these criteria, our final study population comprised 19,799 participants (Figure 1).

### 2.2 Exposure and outcome definitions

Since 2007, NHANES has incorporated kidney stone-related information into its questionnaire. Participants who responded “yes” to the question “Have you/Has sample person (SP) ever had kidney stones?” were identified as having a history of kidney stones.

We adopted a scoring method from previous research (Zhang et al., 2022) for the Oxidative Balance Score (OBS), which comprises 16 dietary and 4 lifestyle components. Among these, 15 are classified as antioxidants and 5 as pro-oxidants. The dietary data was derived from NHANES’ 24-h dietary recall. Lifestyle factors included physical activity, alcohol consumption, body mass index, and cotinine levels. Physical activity was quantified as a metabolic equivalent (MET) score. Alcohol consumption was categorized into three groups: nondrinkers, non-heavy drinkers, and heavy drinkers. Serum cotinine, a metabolite of nicotine, was used as an indicator of exposure to tobacco smoke, encompassing both active and passive smoking. All factors, except for alcohol, were divided into gender-specific tertiles. Antioxidant factors were scored as 0, 1, and 2 from the lowest to highest tertiles, while pro-oxidant factors were scored inversely, from 2 to 0, across the same tertiles. The total OBS score, the sum of all factor scores, indicates a better oxidative balance when higher. Table 1 presents the classification and assigned scores for each OBS component.

**TABLE 1:** Oxidative Balance Score assignment scheme.

OBS components	Property	Male			Female		
		0	1	2	0	1	2
Dietary OBS							
Dietary fiber (g/d)	A	<12.40	12.40–20.50	≥20.50	<10.70	10.70–17.40	≥ 17.40
Carotene (RE/d)	A	<37.01	37.01–135.01	≥135.01	<35.92	35.92–154.38	≥ 154.38
Riboflavin (mg/d)	A	<1.65	1.65–2.50	≥2.50	<1.30	1.30–1.97	≥ 1.97
Niacin (mg/d)	A	<21.37	21.37–31.99	≥31.99	<15.28	15.28–22.97	≥ 22.97
Total folate (mcg/d)	A	<307.00	307.00–483.00	≥483.00	<241.00	241.00–380.00	≥ 380.00
Calcium (mg/d)	A	<684.00	684.00–1123.00	≥1123.00	<582.00	582.00–941.00	≥ 941.00
Zinc (mg/d)	A	<9.06	9.06–13.93	≥13.93	<6.73	6.73–10.26	≥ 10.26
Magnesium (mg/d)	A	<251.00	251.00–362.00	≥362.00	<205.00	205.00–295.00	≥ 295.00
Copper (mg/d)	A	<1.00	1.00–1.46	≥1.46	<0.82	0.82–1.23	≥ 1.23
Selenium (mcg/d)	A	<96.30	96.30–143.30	≥143.30	<70.50	70.50–106.50	≥ 106.50
Iron (mg/d)	P	≥17.61	11.71–17.61	<11.71	≥ 13.73	8.94–13.73	<8.94
Total fat (g/d)	P	≥101.30	65.71–101.30	<65.71	≥ 77.85	49.63–77.85	<49.63
Vitamin B6 (mg/d)	A	<1.62	1.62–2.49	≥2.49	<1.19	1.19–1.88	≥ 1.88
Vitamin B12 (mcg/d)	A	<3.13	3.13–5.99	≥5.99	<2.20	2.2 0–4.22	≥ 4.22
Vitamin C (mg/d)	A	<30.70	30.70–95.60	≥95.60	<29.80	29.80–86.40	≥ 86.40
Vitamin E (ATE) (mg/d)	A	<5.67	5.67–9.56	≥9.56	<4.78	4.78–8.08	≥ 8.08
Lifestyle OBS							
Physical activity (MET-minute/week)	A	<387.50	387.50–1380.00	≥1380.00	<225.00	225.00–720.00	≥720.00
Body mass index (kg/m2)	P	≥30.04	25.70–30.04	<25.70	≥ 31.3	25.2–31.3	<25.2
Alcohol (g/d)	P	≥30	0–30	None	≥15	0–15	None
Cotinine (ng/mL)	P	≥0.95	0.02–0.95	<0.02	≥ 0.09	0.01–0.09	<0.01

A stood in for the antioxidant, P for the pro-oxidant.OBS, oxidative balance score; RE, retinol equivalent; ATE, alpha-tocopherol equivalent;MET, metabolic equivalent.

### 2.3 Covariates

We included the following variables in our research: Age, Race (categorized as Non-Hispanic White, Non-Hispanic Black, Mexican American and Other Race), Marital Status (classified as Married or Living with Partner, and Single), Poverty Income Ratio (PIR) (grouped as<=1.3, 1.3–3.5, >3.5), Education Level (divided into Less than High School, High School Grad/GED or Equivalent, and More than High School), Total Energy Intake, and the presence or absence of Hyperlipidemia, Hypertension, Diabetes, and Gout.

### 2.4 Statistical analysis

In line with the NHANES weighting selection guidelines, we utilized the weights derived from the 24-h dietary recall interview for our study sample. Baseline characteristics of the study population were stratified according to quartiles of the Oxidative Balance Score (OBS). Continuous variables were expressed as weighted means (standard errors), and categorical variables were presented as frequencies (weighted percentages). Differences in baseline characteristics across OBS quartiles were calculated using chi-square tests for categorical variables and *t*-tests or one-way analysis of variance for continuous variables.

We constructed three weighted multivariate logistic regression models to evaluate the relationship between OBS and the kidney stones. The *P* for trend value was calculated when OBS was categorized into quartiles. Model 1 was unadjusted; Model 2 adjusted for age, gender, and race; and Model 3 further adjusted for marital status, poverty income ratio (PIR), education level, energy intake, diabetes mellitus, hypertension, hyperlipidemia, and gout.

Separate analyses were conducted to calculate the associations between dietary OBS scores, lifestyle OBS scores, and the risk of kidney stones. Subgroup analyses were performed based on age, gender, race, marital status, PIR, education level, diabetes, hypertension, hyperlipidemia, and gout to explore potential variations in the relationship between OBS and kidney stones across different subgroups.

A sensitivity analysis was conducted by sequentially excluding each component of the OBS to assess the robustness of our findings (Table 2). All statistical analyses were performed using R version 4.2.2, and statistical significance was determined at a two-sided *p*-value of 0.05.

**TABLE 2:** Sensitivity analyses to assess the effects of individual OBS components on the kidney stone.

OBS	Kidney stone	
	OR (95% CI)	p-value
OBS original model 3	0.97 (0.96,0.99)	<0.001
OBS excluding dietary fiber	0.97 (0.95,0.98)	<0.001
OBS excluding carotene	0.97 (0.95,0.98)	<0.001
OBS excluding riboflavin	0.97 (0.95,0.99)	<0.001
OBS excluding niacin	0.97 (0.95,0.98)	<0.001
OBS excluding vitamin b6	0.97 (0.95,0.98)	<0.001
OBS excluding total folate	0.97 (0.95,0.99)	<0.001
OBS excluding vitamin b12	0.97 (0.95,0.99)	<0.001
OBS excluding vitamin c	0.97 (0.95,0.99)	<0.001
OBS excluding vitamin e	0.97 (0.95,0.98)	<0.001
OBS excluding calcium	0.97 (0.95,0.98)	<0.001
OBS excluding magnesium	0.97 (0.95,0.99)	<0.001
OBS excluding zinc	0.97 (0.95,0.99)	<0.001
OBS excluding copper	0.97 (0.95,0.98)	<0.0001
OBS excluding selenium	0.97 (0.95,0.99)	<0.001
OBS excluding total fat	0.97 (0.96,0.99)	<0.001
OBS excluding iron	0.97 (0.96,0.99)	<0.001
OBS excluding physical activity	0.97 (0.95,0.99)	<0.001
OBS excluding alcohol	0.97 (0.95,0.98)	<0.001
OBS excluding body mass index	0.97 (0.96,0.99)	0.001
OBS excluding cotinine	0.97 (0.96,0.99)	<0.001

Model 3 adjusted for age, gender, race marital status, poverty income ratio (PIR), education level, energy intake, diabetes mellitus, hypertension, hyperlipidemia, and gout.

## 3 Results

### 3.1 Baseline characteristics

In this study, a total of 19,799 participants from the NHANES (2007–2018) were included, with a weighted mean age of 46.3 (0.29) years, and 51.16% being male. The basic demographic characteristics and covariates of the population, stratified by OBS score quartiles, are presented in Table 3. Notably, the highest quartile of OBS had a significantly higher proportion of female participants compared to the other three quartiles. Participants with higher OBS scores were more likely to be Non-Hispanic White, more educated, had a higher PIR, and a higher energy intake. Interestingly, individuals who were single tended to have lower OBS scores. As OBS scores increased (from Q1 to Q4), there was a decrease in the prevalence of hypertension, diabetes, hyperlipidemia, and gout in the population, paralleled by a decrease in the prevalence of kidney stones (7.59% in Q4 vs. 10.27% in Q1, *p* = 0.02).

**TABLE 3:** Basic characteristics of participants by Oxidative Balance Score quartile.

	Oxidative balance score					p-value
Characteristics	Total *N* = 19799	Q1 *N* = 5559	Q2 *N* = 4447	Q3 *N* = 5620	Q4 *N* = 4173	
Age (years)	46.27 (0.29)	45.59 (0.44)	46.86 (0.41)	46.36 (0.34)	46.31 (0.47)	0.1
Gender, n(%)						0.02
Male	10,129 (51.16)	2960 (51.33)	2334 (51.08)	2859 (51.23)	1976 (47.54)	
Female	9670 (48.84)	2599 (48.67)	2113 (48.92)	2761 (48.77)	2197 (52.46)	
Race, n(%)						< 0.0001
Non-Hispanic White	8621 (43.54)	2239 (63.49)	1917 (67.40)	2493 (69.15)	1972 (73.26)	
Non-Hispanic Black	3,926 (19.83)	1550 (15.68)	906 (10.59)	973 (8.63)	497 (5.39)	
Mexican American	2854 (14.41)	691 (7.96)	664 (8.61)	859 (8.52)	640 (7.84)	
Other race	4,398 (22.21)	1079 (12.87)	960 (13.40)	1295 (13.69)	1064 (13.52)	
Marital, n(%)						< 0.0001
Married or Living with partner	11,981 (60.54)	3,026 (55.73)	2691 (63.17)	3,538 (65.28)	2726 (68.41)	
Single	7810 (39.46)	2530 (44.27)	1754 (36.83)	2082 (34.72)	1444 (31.59)	
Family PIR						< 0.0001
≤1.3	5360 (29.52)	1932 (29.33)	1208 (20.14)	1345 (17.51)	875 (14.74)	
1.3–3.5	6730 (37.06)	1948 (36.42)	1528 (35.33)	1940 (34.32)	1314 (29.23)	
>3.5	6069 (33.42)	1224 (34.25)	1327 (44.53)	1839 (48.18)	1679 (56.03)	
Energy (kcal)	2082.44 (7.56)	1537.69 (10.04)	1915.09 (14.26)	2291.37 (14.38)	2534.54 (15.43)	< 0.0001
Education, n(%)						< 0.0001
Less than high school	1597 (8.07)	518 (4.95)	402 (4.25)	423 (3.64)	254 (2.59)	
High School Grad/GED or Equivalent	6812 (34.43)	2378 (41.34)	1575 (32.82)	1823 (29.01)	1036 (21.19)	
More than high school	11,376 (57.5)	2658 (53.70)	2468 (62.92)	3,372 (67.35)	2878 (76.23)	
Hyperlipidemia						< 0.0001
Yes	13,787 (69.63)	4,048 (71.90)	3,166 (71.18)	3,861 (67.98)	2712 (64.37)	
No	6012 (30.37)	1511 (28.10)	1281 (28.82)	1759 (32.02)	1461 (35.63)	
Hypertension						< 0.0001
Yes	7705 (38.92)	2444 (38.24)	1780 (37.11)	2136 (34.27)	1345 (28.61)	
No	12,093 (61.08)	3,115 (61.76)	2667 (62.89)	3,483 (65.73)	2828 (71.39)	
Diabetes, n(%)						< 0.001
Yes	2326 (11.78)	743 (10.11)	566 (9.46)	634 (8.71)	383 (6.68)	
No	17,424 (88.22)	4,800 (89.89)	3,871 (90.54)	4,967 (91.29)	3,786 (93.32)	
Gout, n(%)						< 0.0001
Yes	818 (4.13)	284 (4.49)	195 (4.01)	224 (3.72)	115 (2.18)	
No	18,968 (95.87)	5273 (95.51)	4,249 (95.99)	5390 (96.28)	4,056 (97.82)	
Nephrolithiasis, n(%)						0.02
Yes	1768 (8.93)	582 (10.27)	418 (9.93)	456 (8.95)	312 (7.59)	
No	18,031 (91.07)	4,977 (89.73)	4,029 (90.07)	5164 (91.05)	3,861 (92.41)	

GED, general educational development.

### 3.2 Relationship between OBS and kidney stone

We employed three weighted logistic regression models to investigate the association between OBS and the risk of kidney stones, as presented in Table 4. Across all three models, we observed a protective influence of higher OBS scores on the risk of kidney stones (*p* < 0.05). In Model 3, which accounted for a variety of confounding factors, the findings indicated that with each unit increase in OBS, the risk of kidney stones diminished by 3% (95% CI 0.96–0.99; *p* < 0.001). This negative correlation persisted even after categorizing OBS, with the *P* for trend in Models 1, 2, and 3 being 0.002, <0.001, and 0.002, respectively. In Model 3, when using the lowest quartile OBS group as a reference, the risk of kidney stones in the highest quartile group was reduced by 33% (95% CI 0.50–0.89; *p* = 0.002).Additionally, we investigated the associations of both dietary OBS and lifestyle OBS with the risk of kidney stones (Table 5). A persistent negative correlation between both dietary and lifestyle OBS and the risk of kidney stones was consistently observed across all three models (All *p*-values <0.05).

**TABLE 4:** The relationship between oxidative balance score and kidney stone.

Kidney stone	OR (95% CI); p-value					
	Model 1		Model 2		Model 3	
Continuous	0.98 (0.97,0.99)	<0.001	0.98 (0.97,0.99)	<0.0001	0.97 (0.96,0.99)	<0.001
Q1 (5,15)	References		References		References	
Q2 (15,20)	0.96 (0.78,1.19)	0.72	0.91 (0.73,1.14)	0.4	0.93 (0.74,1.17)	0.52
Q3 (26,26)	0.86 (0.71,1.03)	0.11	0.81 (0.67,0.98)	0.03	0.79 (0.64,0.97)	0.03
Q4 (26,37)	0.72 (0.56,0.92)	0.01	0.67 (0.52,0.87)	0.002	0.67 (0.50,0.89)	0.01
P for trend		0.002		<0.001		0.002

OR, odds ratio;CI, confidence intervals.

Model 1 was unadjusted.

Model 2 adjusted for age, gender, and race.

Model 3 adjusted for age, gender, race, marital status, poverty income ratio (PIR), education level, energy intake, diabetes mellitus, hypertension, hyperlipidemia, and gout.

**TABLE 5:** Association between dietary/lifestyle OBS with kidney stone.

	Kidney stone, OR (95%CI)	*p*-value
Dietary OBS		
Model 1	0.98 (0.97,0.99)	0.005
Model 2	0.98 (0.97,0.99)	0.002
Model 3	0.97 (0.96,0.99)	0.004
Lifestyle OBS		
Model 1	0.94 (0.90,0.98)	0.003
Model 2	0.92 (0.88,0.96)	<0.001
Model 3	0.95 (0.91,0.99)	0.02

OR, odds ratio;CI, confidence intervals.

Model 1 was unadjusted.

Model 2 adjusted for age, gender, and race.

Model 3 adjusted for age, gender, race, marital status, poverty income ratio (PIR), education level, energy intake, diabetes mellitus, hypertension, hyperlipidemia, and gout.

### 3.3 Subgroup analysis

As depicted in Table 6, further stratified analysis revealed that among Non-Hispanic White and Other race groups, a higher OBS was associated with a significant reduction in the risk of kidney stones (*P* for interaction = 0.016). Another variable exhibiting an interaction effect was the level of education (*P* for interaction = 0.031). Among individuals with a higher level of education (more than high school), the relationship between OBS and kidney stones was more pronounced (OR 0.962, 95% CI 0.943, 0.980, *p* < 0.001). In the population without diabetes, it was observed that for each unit increase in OBS, the risk of kidney stones decreased by 3.7% (*p* < 0.0001). However, the association was not significant in the population with diabetes.

**TABLE 6:** Subgroup analysis of the association between oxidative balance score and kidney stone.

Subgroup	Oxidative balance Score [OR (95% CI)]	*p*-value	*p* for interaction
Age			0.452
<50	0.977 (0.958,0.998)	0.028	
≥50	0.975 (0.955,0.995)	0.016	
Gender			0.919
Male	0.968 (0.948,0.988)	0.002	
Female	0.975 (0.953,0.998)	0.034	
Race			0.016
Non-Hispanic White	0.975 (0.957,0.992)	0.006	
Non-Hispanic Black	0.985 (0.955,1.015)	0.315	
Mexican American	0.992 (0.946, 1.041)	0.747	
Other race	0.927 (0.893,0.962)	<0.001	
Marital status			0.778
Married or Living with partner	0.975 (0.957,0.993)	0.008	
Single	0.962 (0.938,0.986)	0.003	
Family PIR			0.767
≤1.3	0.954 (0.927,0.981)	0.001	
1.3–3.5	0.963 (0.941,0.985)	0.001	
>3.5	0.984 (0.960,1.009)	0.200	
Education level			0.031
Less than high school	1.008 (0.946,1.074)	0.802	
High School Grad/GED or Equivalent	0.992 (0.970,1.015)	0.498	
More than high school	0.962 (0.943,0.980)	<0.001	
Hyperlipidemia			0.493
Yes	0.977 (0.961,0.994)	0.009	
No	0.956 (0.929,0.984)	0.003	
Hypertension			0.352
Yes	0.978 (0.956,1.001)	0.055	
No	0.967 (0.948,0.987)	0.001	
Diabetes			0.004
Yes	1.023 (0.982,1.066)	0.273	
No	0.963 (0.948,0.978)	<0.0001	
Gout			0.983
Yes	0.968 (0.911,1.028)	0.278	
No	0.971 (0.955,0.986)	<0.001	

Age, gender, race, marital status, poverty income ratio (PIR), education level, energy intake, diabetes mellitus, hypertension, hyperlipidemia, and gout were adjusted.PIR, poverty income ratio;GED, general educational development.

## 4 Discussion

Using data from the NHANES 2007–2018, our study provides new insights into the relationship between the Oxidative Balance Score (OBS) and the risk of kidney stones. We consistently found an inverse association between OBS and the risk of kidney stones, suggesting that a higher antioxidant and lower pro-oxidant exposure, as indicated by a higher OBS, could potentially reduce the risk of kidney stone formation. This association remained significant even after adjusting for potential confounders and was consistent across both dietary and lifestyle OBS.

The pathogenesis of kidney stones is multifactorial and intricate, involving a blend of genetic, environmental, and dietary factors (Howles and Thakker, 2020; Siener and Hesse, 2021). Oxidative stress (OS), defined by an imbalance between the production of oxidants and the body’s antioxidant defenses, has been implicated in the formation of kidney stones (Xu et al., 2023). Several studies have highlighted the elevated oxidative stress levels in renal stone patients. For instance, Williams et al. (2016) found that mitochondrial function in monocytes from kidney stone patients was compromised, leading to increased levels of inflammation and oxidative stress. Additionally, research by Ma et al. (2014) indicated that, compared to a healthy control group, erythrocytes from stone patients showed diminished antioxidant protein levels and activity. Furthermore, animal experiments (Huang et al., 2009; Huang and Ma, 2015) have revealed that the increase in intrarenal oxidative stress in rats with kidney stones is due to the enhanced excretion of ROS in the urine. Oxalate and calcium oxalate crystals, common constituents of kidney stones, can induce OS in renal epithelial cells, contributing to stone formation (Umekawa et al., 2004; Zhu et al., 2019; Khan et al., 2021; Khan, 2014; W et al., 2021). Our findings, demonstrating a protective effect of higher OBS against kidney stone formation, align with the current understanding of the role of OS in kidney stone pathogenesis.

While several methods exist to calculate OBS, we adopted the approach described by Zhang, dividing OBS into 16 dietary components (14 antioxidants and 2 pro-oxidants) and 4 lifestyle components (1 antioxidant and 3 pro-oxidants). Numerous studies have identified that some of these dietary antioxidants can inhibit the formation of kidney stones, such as dietary fiber (Ferraro et al., 2020), riboflavin (Williams et al., 2016), zinc (Ma et al., 2014), magnesium (Huang and Ma, 2015), copper (Huang et al., 2009), and selenium (Di et al., 2023). However, the relationship between other antioxidants and kidney stones remains heterogeneous in various studies (Ferraro et al., 2016; Letavernier et al., 2016; Malihi et al., 2016; Ferraro et al., 2017; Jaturakan et al., 2017; Bouderlique et al., 2019; Malihi et al., 2019; Taheri et al., 2019). Regarding lifestyle factors, it is well-established that a higher BMI correlates with an increased risk of kidney stones (Tasian et al., 2017; Aune et al., 2018). Some studies have found that physical activity can, to some extent, reduce the risk of kidney stones (Aune et al., 2018), while others found no such association (Ferraro et al., 2015). Surprisingly, alcohol consumption might lower kidney stone risk, possibly because it increases fluid intake (Wu et al., 2020; Zhu et al., 2022). However, considering the other health risks of alcohol, we do not recommend it as a preventive strategy.

In our subgroup analysis, we found a stronger protective effect of OBS among Non-Hispanic White and Other race groups, as well as among individuals with a higher level of education. These findings suggest that the impact of OBS on kidney stone risk may vary across different population subgroups, possibly due to differences in genetic susceptibility, dietary habits, or other lifestyle factors. A review (Wang et al., 2023) examined health disparities in kidney stone disease concerning race, ethnicity, socioeconomic status, and place of residence, and found that white males had the highest risk of kidney stones. A study based on the NHANES database (Feng et al., 2021) found that males, non-Hispanic white people, and obese individuals had a higher prevalence of kidney stones, and the prevalence in females showed a catching-up trend. Research has found that education level and lifestyle play a significant role in the formation of kidney stones (Barghouthy et al., 2021). A higher level of education predicts a relatively higher income level and may be associated with healthier dietary and lifestyle habits. However, the underlying mechanisms for these differences warrant further investigation.

Additionally, we observed no significant link between OBS and kidney stones in diabetic individuals. This could be due to the complex interplay between diabetes, OS, and kidney stone formation. Oxidative stress (OS) plays a pivotal role in the onset and progression of diabetes (Littlejohns et al., 2020; Crivelli et al., 2021). OS can interfere with the metabolic processes of glucose, such as glycolysis, leading to hyperglycemia (Pietropaolo et al., 2017; Abufaraj et al., 2021), which could potentially overshadow the impact of OBS on kidney stone risk in this population. Alternatively, the metabolic abnormalities associated with diabetes, such as insulin resistance and hyperglycemia, could influence kidney stone formation through mechanisms independent of OS (Tangvarasittichai, 2015).

One of our study’s strengths is the use of a large, nationally representative sample, bolstering the applicability of our findings. Furthermore, the comprehensive measure of OBS, incorporating both dietary and lifestyle components, provides a more holistic assessment of antioxidant and pro-oxidant exposures. However, our study has several limitations. The cross-sectional design precludes causal inferences. The NHANES database lacks specific data on the type of kidney stones, and while calcium oxalate stones are predominant, the absence of this specificity is a limitation. The method used to determine stone recurrence in NHANES might not be entirely accurate. Reliance on self-reported data and the 24-h dietary recall could introduce recall bias, and the study’s conclusions might not be generalizable to all kidney stone patients. Future studies should consider these factors and potentially incorporate oxidative stress biomarkers for more accurate results. Future longitudinal studies are needed to confirm our findings and elucidate the potential mechanisms underlying the observed associations.

## 5 Conclusion

Our analysis of the NHANES 2007-2018 data revealed a significant inverse association between the Oxidative Balance Score (OBS) and risk of kidney stone. This relationship suggests that higher antioxidant and lower pro-oxidant exposures may reduce kidney stone risk. Notably, the protective effect of OBS varied across different population subgroups, with pronounced benefits observed among Non-Hispanic White and Other race groups, and those with higher education levels. However, the association was not significant among diabetic individuals. Further longitudinal studies are essential to validate these findings and explore the underlying mechanisms.

## Data Availability

Publicly available datasets were analyzed in this study. This data can be found here: www.cdc.gov/nchs/nhanes/.
